# Novel agonists for serotonin 5-HT_7_ receptors reverse metabotropic glutamate receptor-mediated long-term depression in the hippocampus of wild-type and Fmr1 KO mice, a model of Fragile X Syndrome

**DOI:** 10.3389/fnbeh.2015.00065

**Published:** 2015-03-12

**Authors:** Lara Costa, Lara M. Sardone, Enza Lacivita, Marcello Leopoldo, Lucia Ciranna

**Affiliations:** ^1^Department of Clinical and Experimental Medicine, University of MessinaMessina, Italy; ^2^Department of Biomedical and Biotechnological Sciences, University of CataniaCatania, Italy; ^3^Department of Pharmacy, University of BariBari, Italy

**Keywords:** 5-HT7, 5-HT7R agonists, hippocampus, mGluR-LTD, Fragile X Syndrome

## Abstract

Serotonin 5-HT_7_ receptors are expressed in the hippocampus and modulate the excitability of hippocampal neurons. We have previously shown that 5-HT_7_ receptors modulate glutamate-mediated hippocampal synaptic transmission and long-term synaptic plasticity. In particular, we have shown that activation of 5-HT_7_ receptors reversed metabotropic glutamate receptor-mediated long-term depression (mGluR-LTD) in wild-type (wt) and in Fmr1 KO mice, a mouse model of Fragile X Syndrome in which mGluR-LTD is abnormally enhanced, suggesting that 5-HT_7_ receptor agonists might be envisaged as a novel therapeutic strategy for Fragile X Syndrome. In this perspective, we have characterized the basic *in vitro* pharmacokinetic properties of novel molecules with high binding affinity and selectivity for 5-HT_7_ receptors and we have tested their effects on synaptic plasticity using patch clamp on acute hippocampal slices. Here we show that LP-211, a high affinity selective agonist of 5-HT_7_ receptors, reverses mGluR-LTD in wt and Fmr1 KO mice, correcting a synaptic malfunction in the mouse model of Fragile X Syndrome. Among novel putative agonists of 5-HT_7_ receptors, the compound BA-10 displayed improved affinity and selectivity for 5-HT_7_ receptors and improved *in vitro* pharmacokinetic properties with respect to LP-211. BA-10 significantly reversed mGluR-LTD in the CA3-CA1 synapse in wt and Fmr1KO mice, indicating that BA-10 behaved as a highly effective agonist of 5-HT_7_ receptors and reduced exaggerated mGluR-LTD in a mouse model of Fragile X Syndrome. On the other side, the compounds RA-7 and PM-20, respectively arising from *in vivo* metabolism of LP-211 and BA-10, had no effect on mGluR-LTD thus did not behave as agonists of 5-HT_7_ receptors in our conditions. The present results provide information about the structure-activity relationship of novel 5-HT_7_ receptor agonists and indicate that LP-211 and BA-10 might be used as novel pharmacological tools for the therapy of Fragile X Syndrome.

## Introduction

Serotonin (5-HT) is a monoamine neurotransmitter controlling several physiological functions among which mood, circadian rhythms, body temperature, food intake and nociception. Seven main families (5-HT_1_ through 5-HT_7_) and at least 14 subtypes of 5-HT receptors have been identified to date (Hannon and Hoyer, 2008; Nichols and Nichols, 2008). In the last decade, an important role of 5-HT_7_ receptors in learning and memory has emerged. Serotonin is released in the hippocampus (one of the brain structures most crucially involved in learning) by fibers arising from raphe nuclei (Segal, 1990; Schmitz et al., 1998). 5-HT_7_ receptors are expressed in the hippocampus and modulate neuronal excitability and synaptic transmission (Costa et al., 2012b). Behavioral studies performed on wild-type animals (Perez-Garcia and Meneses, 2005; Eriksson et al., 2008; Freret et al., 2014; Meneses et al., 2015) and on mice lacking 5-HT_7_ receptors (5-HT_7_ KO) (Sarkisyan and Hedlund, 2009) indicate that 5-HT_7_ receptor activation exerts a pro-cognitive action. In light of this, 5-HT_7_ receptors have recently been proposed as a novel target in cognitive diseases (Matthys et al., 2011; Ciranna and Catania, 2014; Gasbarri and Pompili, 2014).

Fragile X Syndrome (FXS) is the most common form of inherited intellectual disability, frequently associated with mood disorders (Hagerman and Hagerman, 2002; Garber et al., 2008), autism (Harris et al., 2008) and increased susceptibility to seizures (Musumeci et al., 2001). FXS is caused by silencing of the Fmr1 gene coding for Fragile X Mental Retardation Protein (FMRP) (Pieretti et al., 1991), an mRNA binding protein that functions as a regulator of protein translation (Laggerbauer et al., 2001; Bechara et al., 2009). One of the main consequences of FMRP absence is a dysregulation of protein synthesis, leading to altered synapse morphology and synaptic dysfunction (Bhakar et al., 2012). The brain of FXS patients shows abnormal dendrite morphology in cortex and hippocampus, with an overproduction of long, thin and immature dendritic spines (Irwin et al., 2000). Evidence of synapse malfunction came from studies on the Fmr1 gene knockout (Fmr1 KO) mouse, an animal model of FXS that displays typical features resembling those of FXS patients, among which alterations in dendritic spine morphology (Comery et al., 1997; Nimchinsky et al., 2001) increased susceptibility to audiogenic seizures (Musumeci et al., 2000) and cognitive impairment (Bernardet and Crusio, 2006; Dolen et al., 2007). *In vitro* studies on Fmr1 KO mice revealed that the lack of FMRP dysregulates protein translation induced by activation of group I metabotropic glutamate receptors (mGluRs), with an overproduction of proteins involved in AMPA receptor endocytosis (Nakamoto et al., 2007). Consistently, mGluR-mediated long-term depression (mGluR-LTD), a form of LTD mediated by activation of mGlu5 receptors and leading to endocytosis of AMPA receptors, is abnormally enhanced in the hippocampus of Fmr1 KO mice (Huber et al., 2002). In line with the “mGluR” theory of Fragile X Syndrome, pharmacological blockade or reduced expression of group I mGlu receptors were found to rescue cognitive impairment and abnormal behavior in Fmr1 KO mice (Luscher and Huber, 2010; Bhakar et al., 2012).

We have recently found that 5-HT_7_ receptor activation is able to reverse mGluR-LTD and mGluR-mediated endocytosis of AMPA receptors both in wild-type and in Fmr1 KO mice (Costa et al., 2012a). These data suggest that abnormal mGluR-mediated signaling in Fmr1 KO mice can be reversed by serotonin acting through 5-HT_7_ receptors, suggesting that selective 5-HT_7_ receptor agonists might become novel pharmacological tools for FXS therapy.

In the present work, we have studied the effect of LP-211, a selective and brain-penetrant agonist of 5-HT_7_ receptors (Leopoldo et al., 2008; Hedlund et al., 2010) and of novel putative agonists of 5-HT_7_ receptors in view of future preclinical studies on Fmr1 KO mice.

## Materials and methods

### Synthesis of 5-HT_7_ receptor ligands

**Figure 1:**
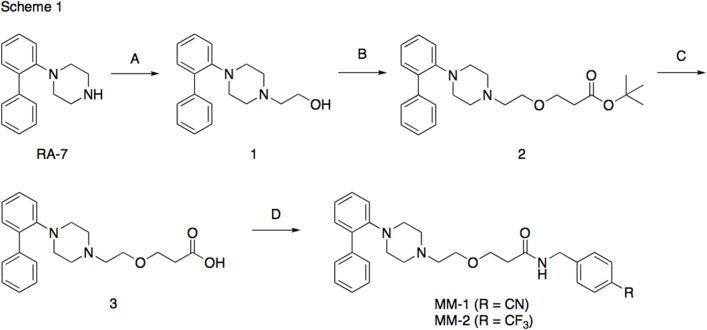
**Synthesis of Compounds MM-1 and MM-2**. Reagents and Conditions: (A) 2-bromoethanol, K_2_CO_3_, KI, acetonitrile, reflux overnight, 82% yield; (B) NaH, *t*-butyl acrylate, anhydrous THF, 0°C to r.t., 20–22 h, 43% yield; (C) 3N HCl, dioxane, r.t., 48 h, quantitative yield; (D) 4-cyanophenylmethylamine or 4-trifuoromethylphenylmethylamine, 1,1'-carbonyldimidazole, anhydrous THF, r.t., overnight, 95% yield.

The synthesis of the following 5-HT_7_ receptor ligands was accomplished as previously reported: *N*-(4-cyanophenylmethyl)-4-(2-diphenyl)-1-piperazinehexanamide, LP-211 (Leopoldo et al., 2008), (*R*)-1-[4-[2-(4-methoxyphenyl)phenyl]piperazin-1-yl]-3-(2-pyrazinyloxy)-2-propanol, BA-10 (Hansen et al., 2014), *N-(2-fluoropyridin-5-ylmethyl)-4-[2-(4-methoxy)phenyl]-1-pip*erazinehexanamide, PF-62 (Lacivita et al., 2014), 1-(2-biphenyl)piperazine, RA-7 (Bantle, 1996), 1-[2-(4-methoxyphenyl)phenyl]piperazine, PM-20 (Lacivita et al., 2012). *N*-(4-cyanophenylmethyl)-3-[4-[2-(4-methoxyphenyl)phenyl]pipe-razin-1-yl]ethoxy]propanamide, MM-1, and *N*-(4-trifuoromethylphenylmethyl)-3-[4-[2-(4-methoxyphenyl)phenyl]pipe-razin-1-yl]ethoxy]propanamide, MM-2, were synthesized as depicted in Figure 1 (Scheme 1). RA-7 was alkylated with 2-bromoethanol to give alcohol 1 that underwent Michael addition to t-butylacrylate to afford the ester 2. Acidic hydrolysis of the ester afforded carboxylic acid 3 that was subsequently condensed with 4-cyanophenylmethylamine or 4-trifuoromethylphenylmethylamine to give the target compounds MM-1 and MM-2, respectively. These synthetic procedures involved purification of intermediate and target compounds by column chromatography on silica gel column. The chemical structure of intermediate and target compounds was confirmed by NMR spectroscopy, mass spectrometry and elemental analysis.

### Evaluation of metabolic stability of novel high-affinity 5-HT_7_ ligands

*In vitro* metabolism of compounds BA-10, PF-62, MM-2, and MM-1 was evaluated after incubation with mouse hepatic microsomes in the presence of an NADPH-generating system, according to Jia and Liu (Jia and Liu, 2007). Test compounds were pre-incubated at 37°C with mouse liver microsomes (0.5 mg/mL microsomal protein) at 1 μM final concentration in 100 mM potassium phosphate buffer (pH 7.4) for 10 min. Metabolic reactions were initiated by the addition of a NADPH regenerating system (NADP, glucose-6-phosphate, glucose-6-phosphate dehydrogenase, final glucose-6-phosphate dehydrogenase concentration, 1 unit/mL). Aliquots were removed (0, 5, 10, 30, and 60 min) and immediately mixed with an equal volume of cold acetonitrile containing internal standard. Test compound incubated with microsomes without NADPH regenerating system for 60 min was included. Quenched samples were centrifugated at 4500 rpm for 15 min and the supernatants were analyzed by reversed phase HPLC. Quantitation of the parent compound at the various time points was performed using Agilent 1290 Infinity LC System equipped with diode array detector. The column used was a Phenomenex Gemini C-18 (250 × 4.6 mm, 5 μm particle size). The samples were isocratically eluted using CH_3_CN/ammonium formate (20 mM, pH 5.5) 7:3 (v/v) as mobile phase. The chromatograms were registered at three different wavelenghts (230, 254, and 280 nm).

Natural logarithm of percentage remaining vs. time data for each compound was fitted to linear regression and the slope was used to calculate the degradation half-life (t_1/2_) according to the equation (Obach et al., 1997):

$${\text{t}}_{1/2}:\frac{0.693}{\text{k}}$$

*In vitro* half-life was then used to calculate the intrinsic plasma clearance (CL_int_) according to the following equation (Obach et al., 1997):

$${\text{CL}}_{\text{int}}\;\frac{0.693}{{\text{t}}_{1/2}}•\frac{\text{volume of incubation }(\mu \text{L})}{\text{protein in the incubation}}$$

### Electrophysiology

Patch clamp experiments were performed on mouse hippocampal slices from wild-type (wt) mice (FVB and C57BL/6J background) and Fmr1 KO2 mice (C57BL/6J background, kindly provided by Prof. Willemsen, Erasmus MC, Rotterdam, The Netherlands). Animal care and experimental procedures were performed in accordance with the European Communities Council guidelines 86/609/EEC. Every care was taken to maximally reduce the number of animals and to minimize discomfort.

Acute hippocampal slices from wt and Fmr1 KO mice (age 14–21 days) were prepared as previously described (Costa et al., 2012a,b). Briefly, the brain was rapidly removed and placed in oxygenated ice-cold artificial cerebro-spinal fluid (ACSF; in mM NaCl 124; KCl 3.0; NaH_2_PO_4_ 1.2; MgSO_4_ 1.2; CaCl_2_ 2.0; NaHCO_3_ 26; D-glucose 10, pH 7.3). Acute hippocampal slices (300 μM) were cut with a vibratome (Leica VT 1200). Slices were continually perfused with oxygenated ACSF. After 3 h of recovery, one slice was placed in the recording chamber of a patch camp set up and viewed with infrared microscopy (Leica DMLFS). A tungsten electrode was placed in the *stratum radiatum* to stimulate Schaffer collaterals with negative current pulses (duration 0.3 ms, delivered every 15 s by A310 Accupulser, WPI, USA). Evoked AMPA receptor-mediated excitatory post-synaptic currents (EPSCs) were recorded from CA1 pyramidal neurons under whole-cell patch clamp (holding potential -70 mV; EPC7-plus amplifier HEKA, Germany). Stimulation intensity was set to induce half-maximal EPSC amplitude. Series resistance (Rs) was monitored throughout the recording by means of 10 mV hyperpolarizing pulses; recordings were discarded from analysis if Rs changed by more than 20%. EPSC traces were filtered at 3 kHz and digitized at 10 kHz. Data were acquired and analyzed using Signal software (CED, England). The recording micropipette (resistance 1.5–3 MΩ) was filled with intracellular solution (in mM: K-gluconate 140; HEPES 10; NaCl 10; MgCl_2_ 2; EGTA 0.2; Mg-ATP 3.5; Na-GTP 1; pH 7.3). Bath solution (ACSF; flow rate of 1.5 ml/min) routinely contained (-)-bicuculline methiodide (5 μM, SIGMA) and D-(-)-2-Amino-5-phosphonopentanoic acid (D-AP5, 50 μM, Tocris). mGluR-LTD of synaptic currents was chemically induced by application of the group I mGluR agonist DHPG (100 μM, 5 min) in the absence and in the presence of a 5-HT_7_ receptor ligand. Pharmacological agents (*S*-3,5-DHPG 100 μM; LP-211 10 nM; BA-10 10 nM; RA-7 10 nM; PM-20 10 nM) were dissolved in ACSF and applied by bath perfusion.

For LTD data analysis, peak amplitude values of evoked EPSCs were averaged over 1 min and expressed as % of control (calculated from EPSCs recorded during at least 15 min before DHPG application). The amount of mGluR-LTD was calculated 45–55 min after LTD induction by DHPG application and is expressed by indicating EPSC amplitude as percentage of baseline EPSC (% EPSC). Cumulative histograms indicate % EPSC amplitude (mean ± SEM from groups of neurons) after application of DHPG alone (control LTD) or DHPG followed by a 5-HT_7_ receptor agonist. EPSC amplitude values from two groups of neurons were compared using the Student's *t*-test, with *n* indicating the number of neurons tested in each condition.

## Results

### The 5-HT_7_ receptor agonist LP-211 reversed mGluR-LTD in wild-type and Fmr1 KO mice

Excitatory post-synaptic currents (EPSCs) mediated by glutamate AMPA receptors were recorded from CA1 pyramidal neurons following stimulation of Schaffer collaterals in hippocampal slices from wt and Fmr1 KO mice (C57BL6J background; post natal day PN 14–21).

**Figure 2:**
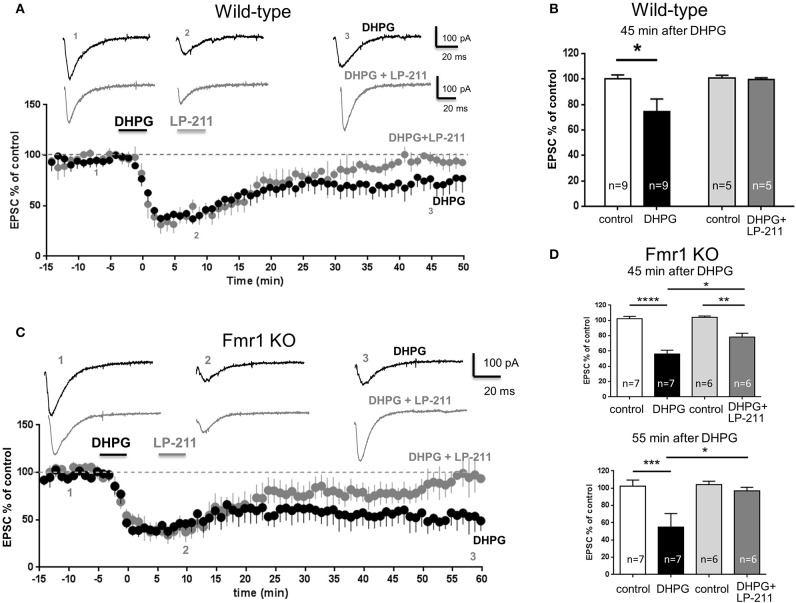
**LP-211 reversed mGluR-LTD in the hippocampus of wild-type and Fmr1 KO mice**. Excitatory post-synaptic currents (EPSCs) mediated by AMPA receptors were recorded under whole-cell patch clamp from CA1 pyramidal neurons after stimulation of Schaffer collaterals in the continuous presence of the NMDA receptor antagonist D-AP5 (50 μM). EPSC amplitude is represented as percent of control EPSC amplitude prior to any substance application. Pooled data of % EPSC values (mean ± SEM) were plotted vs. time; insets show individual EPSC traces from representative experiments. **(A)** Hippocampal slices (300 μm) were prepared from wild-type mice on a C57BL/6J background (PN 14-21). Bath application of DHPG (100 μM, 5 min), an agonist of group I metabotropic glutamate receptors, induced a long term depression of EPSC amplitude. In another group of recordings, LP-211 (10 nM, 5 min) was applied 5 min after LTD induction. Following application of LP-211, EPSC amplitude returned to control values (100%), indicating that mGluR-LTD was reversed. **(B)** Histograms show mean EPSC amplitude values (% of control) in control conditions (empty column, *n* = 9) and 45 min after application of DHPG alone (black column, *n* = 9) or application of DHPG followed by LP-211 (dark gray column, *n* = 5). Application of DHPG significantly reduced EPSC amplitude (^*^P < 0.01), thus induced mGluR-LTD. Vice-versa, when DHPG application was followed by LP-211, EPSC values were not reduced with respect to control, indicating that mGluR-LTD was completely reversed. **(C)** Hippocampal slices were prepared from Fmr1 KO mice (C57BL6J strain; PN 14-21) Application of DHPG (100 μM, 5 min) induced a long-term depression (mGluR-LTD) of EPSC amplitude. When LP-211 (10 nM, 5 min) was applied after DHPG, mGluR-LTD was partially reversed. A complete reversal of mGluR-LTD was observed around 55 min after LTD induction. **(D)**, upper panel: EPSC current amplitude in control conditions (100%) and 45 min after application of DHPG. In slices from Fmr1 KO mice, the amount of mGluR-LTD induced by DHPG alone was highly significant (black column; ^****^P < 0.00001) and was larger than in wild-type (compare with panel **B**). When DHPG application was followed by application of LP-211, the amount of mGluR-LTD was still significant (^**^P < 0.001) but was significantly reduced (^*^P < 0.01) with respect to mGluR-LTD induced by DHPG alone, indicating that mGluR-LTD was partially reversed. **(D)** Lower panel: EPSC current amplitude in control conditions (100%) and 55 min after application of DHPG: mGluR-LTD was completely reversed by LP-211, indicating a slower time course of LP-211-mediated reversal in Fmr1 KO with respect to wild-type.

In slices from wt mice, bath application of DHPG (100 μM, 5 min), an agonist of group I metabotropic glutamate receptors, induced a long term depression (mGluR-LTD) of synaptic currents (Figure 2A): EPSC amplitude measured 45 min after DHPG application (74.4 ± 9.8, mean ± SEM, *n* = 9) was significantly lower than control EPSC amplitude (99.97 ± 3.2, *n* = 9; *P* < 0.01; Figure 2B).

In another group of recordings, LP-211 (10 nM, 5 min) was applied 5 min after DHPG (Figure 2A): in these conditions, EPSC amplitude measured 45 min after DHPG application (99.4 ± 1.5% of control, mean ± SEM, *n* = 5) was not significantly different from control EPSC amplitude (*P* = 0.7, Figure 2B), indicating that mGluR-LTD was completely reversed by LP-211. This result fully confirms our previous data obtained using the FVB mouse strain, showing that LP-211 reversed mGluR-LTD in hippocampal slices from wild-type mice (Costa et al., 2012a).

In slices from Fmr1 KO mice (C57BL6J background), application of DHPG strongly reduced EPSC amplitude (EPSC measured 45 min after DHPG: 56 ± 5% of control, mean ± SEM, *n* = 7; Figure 2C), with a highly significant reduction with respect to control EPSC (*P* < 0.00001, Figure 2D). The amount of DHPG-induced inhibition in slices from Fmr1 KO mice was significantly larger than in wt slices (EPSC amplitude: 74 ± 4% of control, mean ± SEM, *n* = 9; *P* = 0.0069; compare black columns in Figures 2B,D). This result confirms that mGluR-LTD in Fmr1 KO mice was enhanced with respect to wt, in agreement with previous data (Huber et al., 2002; Zhang et al., 2009; Choi et al., 2011; Costa et al., 2012a).

Application of LP-211 after DHPG reversed mGluR-LTD also in slices from Fmr1 KO mice (Figure 2C). Compared to wt, reversal of mGluR-LTD by LP-211 in Fmr1 KO slices had a slower time course: 45 min after DHPG application, the amount of mGluR-LTD was still significant (EPSC amplitude 78 ± 5% of control, mean ± SEM, *n* = 6, *P* = 0.001, Figure 2D, upper panel), but was significantly lower (*P* < 0.01) than that induced by DHPG only. A full reversal of mGluR-LTD by LP-211 was observed 55 min after DHPG application: EPSC amplitude (97 ± 3% of control, mean ± SEM, *n* = 6) was not significantly different from control EPSC (*P* = 0.07; Figure 2D, lower panel).

### *In vitro* pharmacokinetic properties of new putative 5-HT_7_ receptor agonists

The four new putative 5-HT_7_ receptor agonists BA-10, PF-62, MM-2, and MM-1 were identified in our laboratory following a medicinal chemistry campaign to obtain 5-HT_7_ receptor ligands with the same structural scaffold of LP-211 but endowed with improved metabolic stability (Table 1). These molecules incorporated chemical features known to improve metabolic stability, such as electron withdrawing groups, or polar groups that reduce the lipophilicity of the molecule, since it is known that high lipophilicity is correlated with metabolic liability (Mannhold, 2006). The new compounds were initially tested for their binding affinity for 5-HT_7_ receptors. With respect to LP-211, BA-10, and PF-62 displayed an increased affinity for 5-HT_7_ receptors, whereas MM-1 and MM-2 respectively showed comparable and lower affinity (Table 1). This indicated the correct design of the new molecule that was aimed to obtain compounds with the same profile as LP-211. Interestingly, all the compounds tested showed improved selectivity for 5-HT_7_ over 5-HT_1A_ receptors with respect to LP-211 (Table 1).

**Table 1:** **Binding Affinity Data and Pharmacokinetic Parameters of Test Compounds**.

**Compound**	**5-HT_7_*K*_i_ [nM]**	**5-HT_1A_*K*_i_ [nM]**	**t_1/2_ (min)**	**CL_int_ (μL/mg/min)**	**BA/AB**
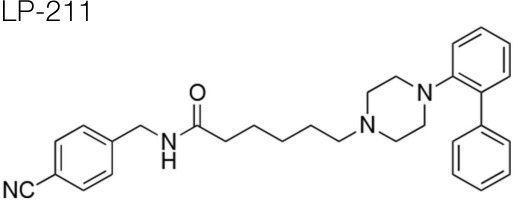	15	188	10.6	131	4.0
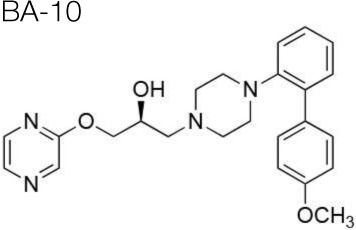	1.1	242	14.8	96	5.6
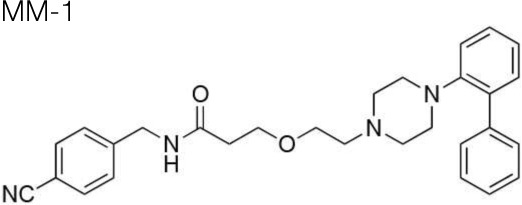	16	437	2.7	515	3.3
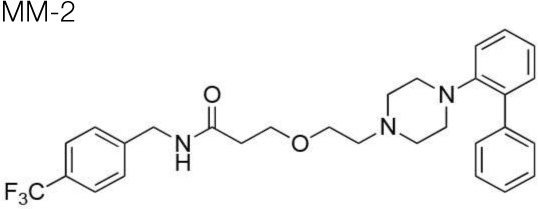	35	582	2.8	498	3.5
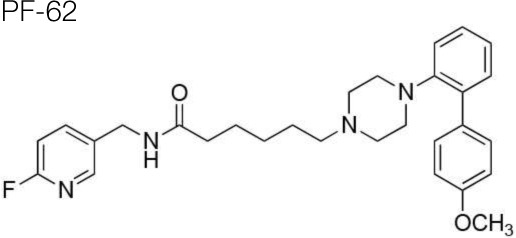	3.82	152	6.7	205	4.2

*In vitro* metabolism of compounds BA-10, PF-62, MM-2, and MM-1 was evaluated after incubation with mouse hepatic microsomes in the presence of an NADPH-generating system, according to Jia and Liu (2007). Liver microsomes provide the most convenient way to study cytochrome P450 enzyme-mediated metabolism. Half-life (t_1/2_) and Intrinsic Clearance (CL_Int_) of the test compounds are listed in Table 1. Half-life and intrinsic clearance of MM-2 and MM-1 indicated that these compounds are less metabolically stable than PF-62, LP-211 and BA-10. Instead, BA-10 appeared endowed with higher metabolic stability than LP-211. In particular, BA-10 displayed the longest *in vitro* half-life among the five compounds examined, indicating improved metabolic stability with respect to LP-211.

We next evaluated the efflux ratio (BA/AB) of PF-62, MM-2, and MM-1 between basal-to-apical (BA) and apical-to-basal (AB) fluxes in Caco-2 cells monolayer. This system is an *in vitro* model to evaluate the interaction of small molecules with P-glycoprotein (P-gp), an efflux pump localized on the blood-brain barrier that prevents xenobiotics to enter the brain. Therefore, a centrally acting drug should not be a P-gp substrate. In this system, a cutoff value of 3 is generally used to distinguish P-gp substrate from nonsubstrate (Hitchcock, 2012). BA/AB ratios of the test compounds are listed in Table 1: all the compounds tested appeared to be weak substrates, thus should only weakly interact with P-gp. In line with this, a recent positron emission tomography study performed with [^11^C]BA-10 clearly showed *in vivo* that BA-10 is not a P-gp substrate, being able to massively accumulate into the brain (Hansen et al., 2014), thus is likely to act on the central nervous system following systemic administration.

In summary, among the new putative 5-HT_7_ receptor agonists examined in the present study, the compound BA-10 displayed improved affinity and selectivity for 5-HT_7_ receptors, together with improved *in vitro* metabolic stability with respect to the 5-HT_7_ receptor agonist LP-211.

### The novel compound BA-10 behaved as an agonist of 5-HT_7_ receptors and reversed mGluR-LTD in the hippocampus of wild-type and Fmr1 KO mice

**Figure 3:**
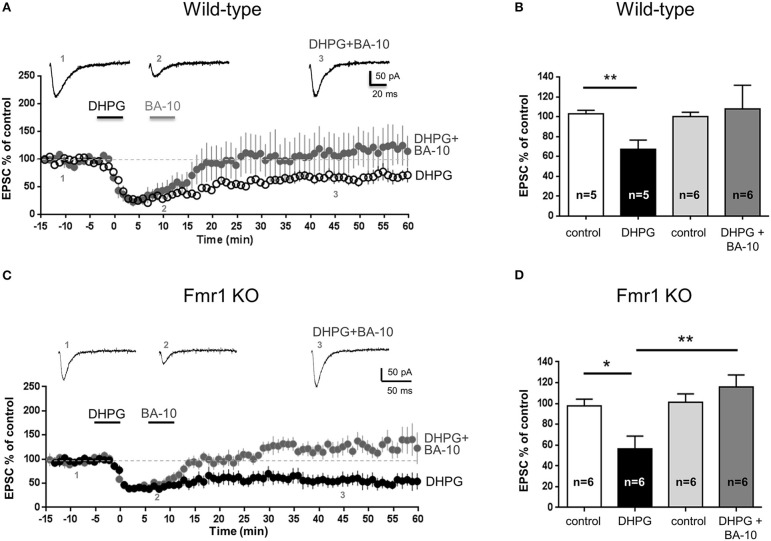
**The novel 5-HT_7_ receptor ligand BA-10 reversed mGluR-LTD in the hippocampus of wild-type and Fmr1 KO mice** Pooled data of % EPSC values (mean ± SEM) were plotted vs. time; insets show individual EPSC traces from representative experiments. mGluR-LTD was chemically induced by bath application of DHPG (100 μM, 5 min). **(A,B)** In hippocampal slices (300 μm) from wild-type mice (FVB; PN 14-21), application of DHPG induced a significant mGluR-LTD (^**^P = 0.007, *n* = 5). When LTD induction was followed by application of BA-10 (10 nM, 5 min), EPSC amplitude was not significantly different from control (*P* = 0.75, *n* = 6), indicating that mGluR-LTD was completely reversed. This result shows that BA-10 behaved as an agonist of 5-HT_7_ receptors. **(C,D)** In slices from Fmr1 KO mice (C57BL6J, PN 14-20), application of DHPG (100 μM, 5 min) induced a significant mGluR-LTD (^*^P = 0.010, *n* = 6) that was completely reversed when BA-10 (10 nM, 5 min) was applied after DHPG (*P* = 0.29, *n* = 6). The amount of LTD induced by DHPG in the absence and in the presence of BA-10 was significantly different (^**^P = 0.0054). This result shows that BA-10 was able to reverse mGluR-LTD in a mouse model of Fragile X Syndrome.

We next tested the biological effect of BA-10 on native 5-HT_7_ receptors in the hippocampus of wild-type mice (PN 14–21). When BA-10 (10 nM, 5 min) was applied after DHPG, EPSC amplitude returned to values comparable to control (EPSC amplitude 45 min after DHPG application: 107 ± 23% of control, mean ± SEM, *n* = 6; Figures 3A,B), indicating that mGluR-LTD was reversed.

In slices from Fmr1KO mice (PN 14–20), application of BA-10 completely reversed DHPG-induced mGluR-LTD (Figure 3C): DHPG induced a significant LTD when applied alone (*P* = 0.01, Figure 3D) but not when applied together with BA-10 (*P* = 0.29, Figure 3D). The amount of mGluR-LTD after application of DHPG alone or DHPG with BA-10 was significantly different (EPSC amplitude respectively 56 ± 12% and 116 ± 11% of control; *P* = 0.0054; Figure 3D).

These results indicate that BA-10, a novel compound with improved pharmacokinetic properties with respect to known 5-HT_7_ receptor agonists, is able to reverse mGluR-LTD in wild-type mice and to rescue synaptic plasticity in Fmr1 KO mice, thus might be envisaged as a new pharmacological tool for Fragile X Syndrome.

### The compounds RA-7 and PM-20 did not modify mGluR-LTD

**Figure 4:**
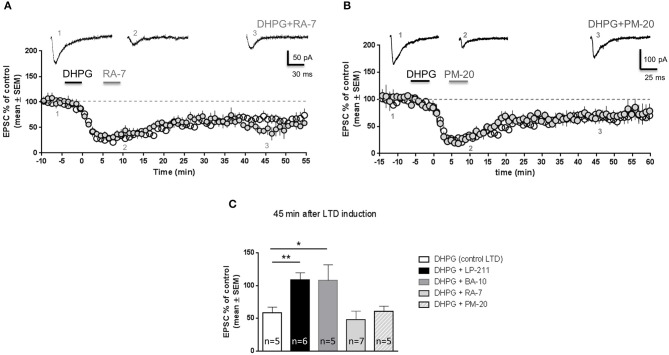
**RA-7 and PM-20 did not reverse mGluR-LTD in the hippocampus of wild-type mice**. Hippocampal slices (300 μm) were prepared from wild-type mice (FVB; PN 14-21). Pooled data of % EPSC values (mean ± SEM) were plotted vs time; insets show individual EPSC traces from representative experiments. When DHPG application was followed by application of RA-7 (**A;** 10 nM, 5 min) or PM-20 (**B;** 10 nM, 5 min), mGluR-LTD was not reversed. **(C)** Histograms show mean EPSC amplitude (% of control) 45 min after DHPG application (empty column, *n* = 5); when DHPG application was followed by application of either LP-211 (black column, *n* = 6) or BA-10 (dark gray column, *n* = 5), EPSC amplitude was significantly higher than following application of DHPG alone (^**^P < 0.001; ^*^P < 0.01 vs. DHPG alone, Student's *t*-test), indicating that mGluR-LTD was reversed. Vice-versa, RA-7 and PM-20 were unable to reverse mGluR-LTD (no significant difference vs. DHPG alone), thus did not behave as agonists of 5-HT_7_ receptors.

Pharmacokinetic studies have shown that LP-211 and BA-10 are metabolized by liver enzymes, respectively giving rise to the compouds RA-7 (Leopoldo et al., 2008) and PM-20 (Prof. Leopoldo, unpublished results). A previous study showed that RA-7 and PM-20 bind 5-HT_7_ receptors with higher affinity than LP-211 (Lacivita et al., 2012). Similar to LP-211, RA-7 is brain penetrant and induced hypothermia in wild-type but not in 5-HT_7_ KO mice following *in vivo* administration (Hedlund et al., 2010). These results suggest that RA-7 might be able to activate 5-HT_7_ receptors. Therefore, we tested the effects of RA-7 on mGluR-LTD. In our experimental protocol, application of RA-7 (10 nM, 5 min) after DHPG application did not reverse mGluR-LTD (Figure 4A): EPSC amplitude measured 45 min after DHPG application was significantly reduced with respect to control (64 ± 7% of control EPSC; mean ± SEM, *n* = 5; P < 0.01). The amount of mGluR-LTD following RA-7 application was not significantly different from mGluR-LTD in control conditions (*P* = 0.5, Figure 4C).

In a similar way, application of PM-20 did not reverse DHPG-induced mGluR-LTD (Figure 4B): EPSC amplitude measured 45 min after DHPG application was significantly lower than control (Figure 4C, EPSC amplitude 60 ± 7% of control, mean ± SEM, *n* = 5; *P* = 0.001). The amount of mGluR-LTD induced by DHPG followed by PM-20 was not significantly different from mGluR-LTD induced by application of DHPG alone (*P* = 0.9, Figure 4C)

These results indicate that RA-7 and PM-20 were unable to induce the same effect of their parent compounds LP-211 and BA-10, thus did not behave as agonists of 5-HT_7_ receptors in our experimental conditions.

## Discussion

In a previous work, we have shown that 5-HT, through activation of 5-HT_7_ receptors, is able to reverse exaggerated mGluR-LTD in the Fmr1 KO mouse model of Fragile X Syndrome (FXS) (Costa et al., 2012a). Exaggerated mGluR-LTD in Fmr1 KO mice is considered an electrophysiological readout of abnormal signaling through mGlu receptors (Bhakar et al., 2012). Our current hypothesis is that activation of 5-HT_7_ receptors, by correcting mGluR-mediated mechanisms in Fmr1 KO mice, besides reversing abnormal mGluR-LTD can also rescue other typical phenotypes of FXS, particularly abnormal dendrite morphology, cognitive impairment and autistic behavior (Osterweil et al., 2012; Ciranna and Catania, 2014). Interestingly, 5-HT_7_ receptor activation was shown to stimulate neurite and dendrite outgrowth in cultured neurons and is believed to play a crucial role in brain wiring during development (Volpicelli et al., 2014). These data suggest that activation of 5-HT_7_ receptors might also correct abnormal dendrite morphology in Fmr1 KO mice.

To test this hypothesis, selective and high-affinity 5-HT_7_ receptor agonists are needed: most importantly, the new agonists must also display drug-like properties and reach the brain following *in vivo* systemic administration. On this purpose, in the present work we have tested the effects of a brain-permeant 5-HT_7_ receptor agonist (LP-211) and characterized the pharmacokinetic properties and biological effects of a novel putative 5-HT_7_ receptor agonist (BA-10). We show that both substances are able to correct a synaptic malfunction in Fmr1 KO mice, thus can be used for *in vivo* administration to Fmr1 KO mice in preclinical behavioral studies.

LP-211 was designed and synthesized by the research group of Prof. Leopoldo (compound 25 in (Leopoldo et al., 2008) and characterized as a high-affinity, selective and brain-permeant 5-HT_7_ receptor agonist (Hedlund et al., 2010). *In vivo* administration of LP-211 was shown to exert pro-cognitive effects in rats submitted to an autoshaping learning task (Meneses et al., 2015).

Our previous results (Costa et al., 2012a) demonstrated that LP-211 is able to reverse mGluR-LTD in wild-type mice. Here we show that LP-211 reverses mGluR-LTD also in Fmr1KO mice, in which mGluR-LTD is abnormally enhanced, suggesting a potential therapeutic use of LP-211 in Fragile X Syndrome.

In slices from Fmr1KO mice, reversal of mGluR-LTD by LP-211 showed a slower time-course with respect to wt. In addition we observed that, unlike in wt, in Fmr1 KO mice LP-211 did not fully reverse mGluR-LTD but rather restored LTD level comparable to wt condition. This result is in agreement with our previous observation that in Fmr1 KO mice 8-OH-DPAT partially reduced the amount of mGluR-LTD and did not completely abolish it (Costa et al., 2012a). This might have important functional consequences, since long-term synaptic plasticity plays a fundamental role in shaping the structure and function of brain circuits. A large body of evidence demonstrates that LTD is crucially involved in learning and memory and pharmacological or genetic manipulations disrupting LTD also impair learning (Collingridge et al., 2010). Consistently, abnormal mGluR-LTD has been observed in animal models of several cognitive diseases including Fragile X Syndrome (D'Antoni et al., 2014). Interestingly, LTD plays a crucial role in novelty detection and in the extinction of previously acquired memories and is believed to underlie behavioral flexibility (Collingridge et al., 2010). A reduced behavioral flexibility, i.e., a reduced ability to face a new environmental context, is a typical feature of autism spectrum disorders. Fragile X Syndrome is a leading genetic cause of autism (Hagerman et al., 2005) and alterations in behavioral flexibility have been described in FXS patients (Hooper et al., 2008) as well as in Fmr1KO mice (Bernardet and Crusio, 2006; Casten et al., 2011; Krueger et al., 2011). We suggest that selective activation of 5-HT_7_ receptors, by restoring mGluR-mediated synaptic plasticity to normal levels, might also rescue cognitive functions and behavioral flexibility in the mouse model of Fragile X Syndrome (Ciranna and Catania, 2014). We will test this hypothesis in the near future by behavioral studies on wt and Fmr1 KO mice following *in vivo* administration of a 5-HT_7_ receptor agonist.

As already pointed out, LP-211 is suitable as a centrally-active substance following systemic administration, being able to pass the blood brain barrier (Hedlund et al., 2010), but showed a relatively short *in vivo* half-life (65 min) when administered to mice by intraperitoneal injection (Leopoldo et al., 2008). In the attempt to identify a 5-HT_7_ receptor agonist with improved pharmacokinetic properties, we have designed and characterized a series of new molecules structurally related to LP-211.

Among the novel compounds examined in the present work, BA-10 displayed the most suitable pharmacokinetic properties for systemic administration. Vice-versa, the compounds MM-1 and MM-2 displayed lower *in vitro* metabolic stability compared to LP-211 and were not further considered for our study. As for compound PF-62, *in vitro* pharmacokinetic properties did not show any improvement with respect to LP-211. On the other side, BA-10 displayed higher selectivity on 5-HT_7_ receptors and improved metabolic stability with respect to LP-211. We show that BA-10 significantly reversed mGluR-LTD (an effect mediated by 5-HT through activation of 5-HT_7_ receptors, as we have previously characterized) in the hippocampus of wild-type and Fmr1 KO mice. These results demonstrate that BA-10 behaves as an agonist of 5-HT_7_ receptors and is able to correct exaggerated mGluR-LTD in the mouse model of Fragile X Syndrome. In slices from Fmr1 KO mice, reversal of mGluR-LTD by BA-10 at a 10 nM dose was more significant than the effect induced by LP-211 at the same concentration (compare Figures 2C,D and Figures 3C,D). The higher effectiveness of BA-10 with respect to LP-211 is in agreement with a 10 fold higher affinity of BA-10 vs. LP-211 for 5-HT_7_ receptors, as we measured by radioligand binding assays (see Table 1), and indicates that BA-10 can be used at very low (sub-nanomolar) doses to activate 5-HT_7_ receptors.

We observed that BA-10 showed a weak *in vitro* affinity for interaction with P-glycoprotein, suggesting that BA-10 might persist in CNS without being extruded by the blood brain barrier when administered systemically. Consistently, *in vivo* results recently published (Hansen et al., 2014) show that BA-10 is not significantly transported by P-glycoprotein and reaches high brain concentrations following intravenous injection. Therefore, the pharmacokinetic properties of BA-10 are promising and deserve to be further investigated.

RA-7 and PM-20, metabolites of LP-211 and BA-10 respectively, were unable to reverse mGluR-LTD, thus did not behave as agonists of 5-HT_7_ receptors in our experimental protocol. This indicates that, following *in vivo* administration of LP-211 and BA-10, their metabolites RA-7 and PM-20 do not exert a cooperative action with parent compounds, as was hypothesized for RA-7 (Hedlund et al., 2010). The lack of agonist effect of RA-7 and PM-20 also suggests that, following *in vivo* administration, the effects of LP-211 and BA-10 are likely to be reduced with time as their concentration is decreased by hepatic metabolism. Nevertheless, several results indicate that LP-211 exerts long-term effects on the brain. The first pharmacokinetic study on LP-211 (Leopoldo et al., 2008) shows that the brain concentration of LP-211 in mice remained high during at least 2 h after intraperitoneal injection. The authors suggested that LP-211 is likely to be accumulated in the brain, due to its high lipophilic properties. Besides, it should be noticed that 2 h after injection, LP-211 brain concentration declined below detectable levels, but might still be high enough to activate 5-HT_7_ receptors, especially considering that LP-211 binds 5-HT_7_ receptors with very high affinity, with reported Ki values between 0.58 nM (Leopoldo et al., 2008) and 15 nM (Hedlund et al., 2010). In line with this, we observed a reversal of mGluR-LTD by a low nanomolar dose of LP-211 (10 nM). Consistently, LP-211 has been administered *in vivo* in several studies and was shown to exert long-term effects on functions regulated by the central nervous system such as regulation of body temperature, sleep and circadian rhythms (Hedlund et al., 2010; Adriani et al., 2012; Monti et al., 2014). These results suggest that central effects of LP-211 can be long-lasting in spite of a relatively short half-life.

In conclusion, we show that the 5-HT_7_ receptor agonists LP-211 and BA-10 correct a synaptic malfunction in Fmr1KO mice, thus might become new pharmacological tools for the therapy of Fragile X Syndrome. We also show that the novel compound BA-10 is a highly effective 5-HT_7_ receptor agonist with improved selectivity and *in vitro* pharmacokinetic properties with respect to LP-211, thus should also be considered for *in vivo* studies.

### Conflict of interest statement

The authors declare that the research was conducted in the absence of any commercial or financial relationships that could be construed as a potential conflict of interest.
